# Letter from Strassburg

**Published:** 1882-08

**Authors:** Frederick Peterson

**Affiliations:** Strassburg


### Correspondence.

EDITORS JOURNAL: STRASSBURG, July 1, 1882.

Since my last letter from Vienna, I have been studying in Strassburg, attracted here chiefly by the fame of Von Recklinghausen. It is fast becoming one of the best of German universities. In the eighteenth century it was German, and Goethe was one of its graduates in the law department, but since that time, until ten years ago, it was a French Academy with but few students. After the recent cession of Alsace to Germany, the Berlin Government has paid attention to making the city as much an university town as an impregnable fortress. In addition to many new buildings already complete, are still five or six magnificent structures in process of erection. The medical faculty is a brilliant one. The anatomist and histologist, Waldeyer with the pathologist, Von Recklinghausen, have one building together, with probably the finest laboratories in Europe. In the same building are the anatomical and pathological museums, the former rich in marvelous work, from the skilled hands of Waldeyer and Jössel. For these two branches of medical science, Strassburg is now pre-eminent. No other university has such teachers, such material or such conveniences. Both professors are arduous laborers; from early morning until seven every evening at their posts, and take the greatest pains with their pupils. Von Recklinghausen makes some five or six post mortem examinations daily, which together with material sent him by physicians in the city, furnish sufficient for six hours weekly demonstration. He is considered here as second to Virchow, and it is said he will be called upon to succeed his former teacher in Berlin, upon the decease of the latter, who is already at an advanced age; but it is also said he will not leave Strassburg. The fine laboratory of Hoppe-Seyler, professor of physiological chemistry, is another building in the same neighborhood. Indeed nearly all of the medical department buildings are near together. The name of Kussmaul is so well-known, I need but mention that he is professor here of internal medicines, and to be heard in the Bürgerspital every morning for two hours. The Bürgerspital is a small village in itself. In it are the clinics for internal medicine, diseases of children, eye and ear, psychiatry and the poliklinik. The only one of the old French professors who cared to remain in connection with the university after the late war is Aubenas who still holds forth on *maladies puerperales.*

Koeberle, a star of the French period, famed for his ovariotomies, though still a resident of Strassburg, is no longer a teacher. He has a hospital of his own to which no students are admitted. Through the influence of a Buffalo physician, Dr. Tobie, I have had the pleasure of being invited to operations by Koeberle during my stay in Strassburg, His method of ovariotomy differs from any I have seen before in several ways; in the speed with which he opens the abdomen, in the slowness with which he proceeds afterward, in the care he takes to check the smallest oozing of blood, and in the closing of the abdominal wound. The ligature of the pedicle is of strong Chinese silk. Silk stitches are put through the subperitoneal fibrous coat, leaving the peritoneum wholly intact, and the skin and subcutaneous tissues brought together with an ordinary continued silver pin suture. In this way a very close and even adjustment of the sides of the incision is made, and drainage of the pus around the stitches outward, and outside of the peritoneum secured. His only antiseptic precautions are the placing of the instruments in a mild solution of carbolic acid, and the laying of a little sharpie moistened with the same over the wound. Around the abdomen a simple bandage is pinned. The legs are bound together and a bolster placed under the knees to prevent tension upon the wound. The anæsthetic used is chloroform. The operation is performed in the bed in which the patient is to lie. Freund is professor of midwifery and diseases of women. He also is famed for his ovariotomies, but more especially for his operations for removal of the uterus. He has at present a hospital of his own, but the new woman's hospital is rapidly undergoing construction, near the other medical buildings. Freund is a man of very lucid and pleasing address. Some of the other professors are Goltz on physiology, Schmiedeberg on therapeutics, Jolly on psychiatry, and Laqueur on the eye.

The University library is very important, consisting of 500,000 volumes, with a well selected medical portion, and in the reading room are to be found medical journals in all languages, America being represented by some of her best. Just here I would like to speak of my mortification at finding that the only American medical journals at the reading room of the hospital at Vienna were the *Boston Medical Record** and that contemptible advertiser of Parke, Davis & Co., known as the *New Remedies*,* nearly every article in which glories in the name of that firm. The excellence of the *Boston Medical Record* can scarcely annul the disgrace of having that Detroit journal considered by the Viennese, as to some extent representative of legitimate American medical journalism. Could not the editors of our best journals contribute a copy and send to the *Lesezimmer*, *Allgemeines*, *Krankenhaus*, Vienna?

The recent experiments of Koch in Berlin, resulting in the discovery of the bacteria of tubercle, were carefully repeated by one of the Russian students in the laboratory of Von Recklinghausen, and the slender, blue bacilli shown to the professors and his friends. If within the next few years, it be corroborated that the bacilli are really the cause of tubercle, then it remains but for therapy to discover their destroyer, and the scourge of the world will be annihilated. It looks as if physicians were not to work in the dark much longer.

Living expenses in Strassburg are little lower than in Vienna, and there is a marked difference in the fees for lectures. None of the lecture courses cost over $8 per semester, five months. One can learn most in Vienna in shortest time, but for long stays, undoubtedly Strassburg is much superior.

---

* Our correspondent has certainly made a mistake here in names. We never heard of the *Boston Medical Record*. *New Remedies* is published in New York; in its special field it is an exeellent Journal and one which an American need not be ashamed of.—Editors.

The number students for this semester in the university was 700. It would have been greater, had not a severe epidemic of smallpox during the whole spring until now, frightened many away.

I must now bring this letter, at the same time with my stay in Strassburg, to a close. Perhaps toward autumn I will have something to say of the University of Stockholm, where I go now to study with Axel Key, one of the best of living pathologists, and to whom I carry a message and greetings from Von Recklinghausen, for the two were students together under Virchow.

FREDERICK PETERSON.

